# Tailored Toxicity-Driven Administration of Vismodegib in Patients With Multiple or Locally Advanced Basal Cell Carcinoma: A Pilot Analysis

**DOI:** 10.3389/fonc.2020.563404

**Published:** 2020-11-13

**Authors:** Maria Chiara Tronconi, Alessandra Solferino, Laura Giordano, Riccardo Borroni, Luca Mancini, Armando Santoro

**Affiliations:** ^1^ Medical Oncology and Hematology Unit, Humanitas Cancer Center, Humanitas Clinical and Research Center, IRCCS, Rozzano, Italy; ^2^ Pharmacist Oncology and Hematology Unit, Humanitas Cancer Center, Humanitas Clinical and Research Center, IRCCS, Rozzano, Italy; ^3^ Biostatistics Unit, Humanitas Cancer Center, Humanitas Clinical and Research Center, IRCCS, Rozzano, Italy; ^4^ Dermatology Unit, Humanitas Cancer Center, Humanitas Clinical and Research Center, IRCCS, Rozzano, Italy; ^5^ Department of Biomedical Sciences, Humanitas University, Pieve Emanuele, Italy

**Keywords:** basal cell carcinoma, vismodegib, tailored treatment, toxicity, schedule

## Abstract

In this pilot study, we describe our experience with vismodegib in the treatment of basal cell carcinoma (BCC) and evaluate the feasibility of a tailored toxicity-driven administration of vismodegib in patients with multiple or locally advanced BCC. We retrospectively analyzed the clinical charts of 17 consecutive patients with BCC who were treated with vismodegib. Therapy was started at the usual dosage of 150 mg per day per person, continuously; a rescheduled dosage of 150 mg per day for 4 weeks with a subsequent stop of 2 weeks was allowed during the treatment according to the standard practice of our institution. During treatment, 14 patients with responsive disease presented an adverse event (100% cramps and 20% dysgeusia), therefore, requiring a change in the treatment plan. Overall, in eight out of 17 patients (47% of the overall population), it was possible to re-schedule the treatment by postponing therapy for 2 weeks every 4 weeks. These patients were all still alive at the time of the present analysis and were showing complete response. Adverse events resolved during the first interruption of therapy. The intermittent vismodegib schedule assessed in this pilot series could be beneficial in improving duration of treatment, allowing to maintain a long-term treatment response, even in an elderly and fragile population. Based on these preliminary findings, dedicated studies may be planned to further evaluate an intermittent schedule of vismodegib administration.

## Introduction

Basal cell carcinoma (BCC) remains the most frequently diagnosed cancer worldwide, and its incidence is continuing to increase (1, 2). In most cases, curative treatment with surgery is possible; however, therapy is more challenging when patients present locally advanced or metastatic disease (3). In recent years, the hedgehog signaling pathway has been shown to play a major role in the pathogenesis of BCC (3, 4). Therefore, blocking the hedgehog pathway can represent a suitable strategy in the treatment of advanced BCC.

Vismodegib is an oral inhibitor of hedgehog pathway, approved for locally advanced and metastatic BCC in patients who are not amenable to surgery or radiation (5–9). Some field-practice experiences have evaluated the use of vismodegib for the treatment of BCC in daily practice (10–15). However, the description of a further series of patients will help towards a more comprehensive evaluation of this treatment.

In particular, adverse events (AEs) are not uncommon with the chronic use of vismodegib, especially at the gastrointestinal and musculoskeletal level, and often lead to early interruption of treatment (16–18). This has important clinical consequences because vismodegib, in some cases, does not eradicate the disease, and the tumor may, therefore, regrow when therapy is definitively interrupted.

In the randomized, double-blind, phase II MIKIE trial, two different intermittent schedules of vismodegib demonstrated promising activity in long-term regimens in patients with multiple BCCs, suggesting the needs for future studies on alternative schedules (19). The feasibility of re-treatment with vismodegib was shown in some field-practice experiences (11, 12). Moreover, a recent update of the STEVIE trial, which included 1215 patients, showed that temporary treatment interruptions and no definitive suspension is associated with a better prognosis, although the evaluation of this therapeutic strategy was not the primary objective of the analysis (20). Reformulation of vismodegib schedule, without interrupting therapy, therefore, appears to be a potentially intriguing strategy.

In this pilot study, we describe our experience with vismodegib in the treatment of BCC and we evaluate the feasibility of a tailored toxicity-driven administration of vismodegib in patients with multiple or locally advanced BCC.

## Patients and Methods

### Study Setting and Design

We retrospectively analyzed the clinical charts of 17 consecutive patients with BCC treated with vismodegib referring at Humanitas Clinical and Research Center from May 2015 to January 2019. The local Ethical Committee has approved the study design and all patients signed an informed consent to the use of their data for research purposes.

### Treatment Strategy

Vismodegib was prescribed only in the presence of progressive disease that was not manageable with surgery and/or radiotherapy. Therapy was started at the usual dosage of 150 mg per day per person, continuously; a rescheduled dosage of 150 mg per day for 4 weeks with a subsequent stop of 2 weeks was allowed during the treatment according to the standard practice of our institution.

### Assessments

According to the standard practice of our institution, patients were monitored monthly by clinical evaluation (physical examination and laboratory blood tests). Radiological assessment by computed tomography or magnetic resonance imaging, depending on the site of disease, was performed every 2 months. Tumor response was assessed according to the Response Evaluation Criteria in Solid Tumors (RECIST) 1.1 criteria. Furthermore, complete response (CR) was defined as complete disappearance of all target lesions, partial response (PR) as ≥30% decrease in the sum of the longest diameters of target lesions compared with baseline, progressive disease (PD) as ≥20% increase in the sum of the longest diameters of target lesions compared with baseline. Stable disease (SD) was defined as neither PR or PD. AEs were evaluated at each clinical evaluation and were classified according to Common Terminology Criteria for Adverse Events (CTCAE) 4.03.

### Data Analysis

Data were summarized as number and percentages for categorical data or as median and range for continuous data. No comparisons were performed due to the explorative descriptive nature of the study.

## Results

### Patient Population

The median age of the 17 patients of our series was 78 years (range: 41–89), eight were females (47%); two of them had Gorlin–Goltz syndrome (patients #6 and #11). The majority of lesions (76%) were localized on the head and had an aggressive histology (**Table 1**). Most patients (n=13; 77%) underwent surgery; radiotherapy was administered in two patients. Data of individual patients are reported in **Table 2**.

**Table 1:** Patient characteristics at baseline.

Patient characteristics	n	%
Age (years); median (range)	78 (41–89)	
Gender:		
* Female* * Male*	8 9	47 53
Baseline ECOG performance status: * 0*	6	35
* 1* * 2*	8 3	47 18
Histology:		
* Micronodular* * Ulcus Rodens* * Desmoplastic*	8 5 4	47 29 24
Disease site:		
* Head* * Inferior legs* * Back* * Body and Head*	13 1 1 2	76 6 6 12
TNM:		
* T3N0M0* * T4M0N1*	16 1	94 6
Comorbidities	10	59
Gorlin–Goltz syndrome	2	12
Major axis (cm); median (range)	1.5 (0.3–10)	
First treatment:		
* Surgery* * Radiotherapy + surgery* * Photodynamic + surgery* * None*	13 2 1 1	77 12 6 6

**Table 2:** Data of individual patients.

ID	Age (years)	Age at diagnosis (years)	Gender	Comorbidities	BCC history	Histology	Age at initiation of vismodegib (years)	First response	Reformulation in schedule	Decrease in number of BCCs	Decrease in tumor size	Healing of ulcerations	Adverse events	Status	Survival (months)
1	77	30	F	Kidney cancer, osteoarthritis	Multiple BCC, body, not Gorlin	Micronodular	74	PR	Yes	Yes	Yes	Yes	Creatinine increase	Alive	22.1
2	69	64	M	ci–rrhosis, hypertension	Single BCC, lower left eyelid	Ulcus rodens	64	SD	No	No	Unmeasurable disease		Transaminitis	Alive	45.3
3	86	75	F	Ischemic heart disease, atrial fibrillation, hypertension	Single BCC, nasal pyramid	Desmoplastic	84	PR	No	No	Yes	Yes	Weight loss	Dead	6.8
4	85	74	F	No	Single BCC, left temporal region	Desmoplastic	81	CR	No	No	Yes	Yes	Weight loss	Alive	27.3
5	78	68	M	Hypertension, gastritis	Single BCC, left nose–ocular angle	Ulcus rodens	74	CR	Yes	No	Yes	Yes	Cramps	Alive	30.7
6	43	31	M	No	Multiple BCC, body, Gorlin	Micronodular	41	CR	Yes	Yes	Yes	Yes	Cramps	Alive	14.5
7	91	88	M	Anemia, atrial fibrillation	Multiple BCC, face, not Gorlin	Micronodular	88	PR	No	Yes	Yes	Yes	Weight loss	Dead	5.7
8	82	71	F	No	Single BCC, right nasal wing	Desmoplastic	79	CR	Yes	No	Yes	Yes	Cramps	Alive	21.1
9	81	74	F	No	Multiple BCC, face, not Gorlin	Micronodular	77	PR	No	Yes	Yes	Yes	Cramps	Alive	35.2
10	83	78	M	Hypertension, cognitive decay	Multiple BCC, face, not Gorlin	Ulcus rodens	79	SD	No	No	Yes	Yes	Disgeusia	Alive	36.3
11	87	32	F	No	Multiple BCC, Gorlin	Ulcus rodens	83	CR	Yes	Yes	Yes	Yes	Cramps	Alive	24.5
12	80	77	M	No	Multiple BCC of the massive facial extensively infiltrating eyeballs, not Gorlin	Micronodular	78	SD	No	No	No	No	Infection	Dead	7.8
13	56	22	M	No	Single BCC, right nasal wing	Micronodular	53	CR	No	No	Yes	Yes	Transaminitis	Alive	10.8
14	72	68	M	No	Single BCC, right nasal wing	Micronodular	70	SD	No	No	Yes	Yes	Disgeusia	Alive	9.6
15	54	50	F	No	Single right lower eyelid BCC	Micronodular	52	CR	Yes	No	Yes	Yes	Cramps	Alive	7.4
16	90	58	M	No	Multiple BCC, trunk and lower limbs, not Gorlin	Desmoplastic	89	PR	Yes	Yes	Yes	Yes	Cramps	Alive	3.2
17	87	85	F	Hypertension, vasculopathy, diabetes	Single BCC, left inferior leg	Ulcus rodens	85	PR	Yes	No	Yes	Yes	Weight loss	Alive	13.2

The indication to activate vismodegib in most cases was represented by the presence of multiple tumors or a single tumor that was already treated over the years with different loco-regional treatments. In four patients (patients #2, #8, #13, and #14), vismodegib therapy was started due to the diffuse microscopic presence of BCC in histopathological specimens, not susceptible to further surgery or local therapies (e.g. radiotherapy). In one case (patient #9), there was a nodal loco-regional involvement. No metastatic patients were included in the present analysis.

### Response Before Changing the Treatment Plan

At the first evaluation (after 2 months), disease control was achieved in all 17 patients, showing both clinical and esthetic benefit. Furthermore, seven patients (42%) reported a complete response, six patients (35%) reported a partial response and four patients (23%) had stable disease. However, two patients (12%) progressed soon after the first assessment and one patient (6%) interrupted treatment after 6 months for progressive disease.

The two patients (patients #10 and #12) who progressed immediately after the first assessment experienced esthetic and functional benefit. They presented a long history of disease, with recent rapid destructive and disabling progressions. The other two patients with stable disease (patients #2 and #14) had no measurable disease (microscopically infiltrated margins by BCC); there is still no evidence of relapse at the latest follow-up for patient #14.

In most cases, there was a measurable clinical response. In the two cases (patient #2 and patient #14) of stable disease, it was not possible to measure disease as per the RECIST Criteria and no change of the tumor area was evident after dermatological examination. In other two cases of multiple BCC of the face (patients #10 and #12), vismodegib maintained the overall response; periodic iconographic documentation helped define the stable disease of these patients (never exceeding 10% of initial diameters). In three cases of clinical complete remission of BCC (patients #5, #8, and #13), we performed random biopsies of tumor areas with negative results for persistence of BCC.

### Vismodegib Retreatment

During the treatment, 14 patients with responsive disease presented an AE (100% cramps, and 20% dysgeusia), thus requiring a change in the treatment plan.

**Figure 1:**
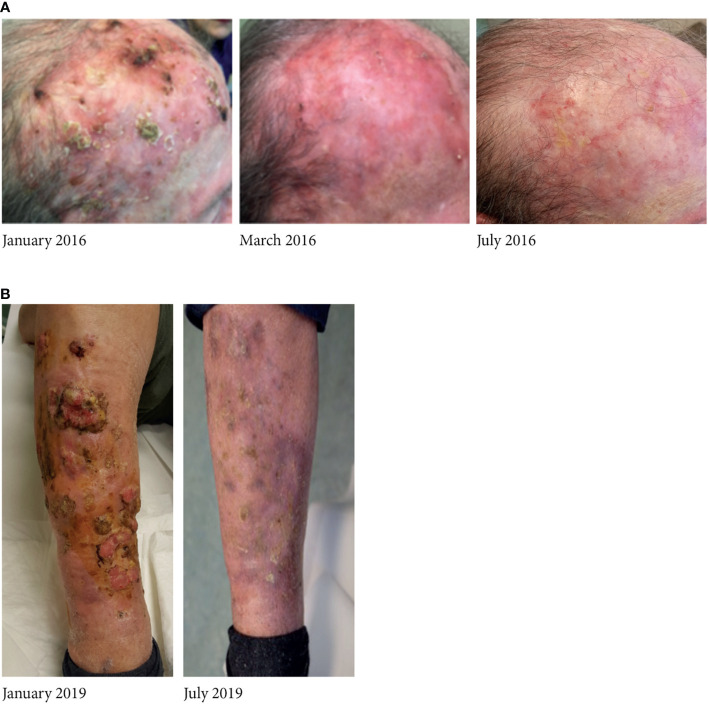
A relevant case of complete response to vismodegib in **(A)** an 83-year-old woman and in **(B)** an 86-year-old man, who continued treatment with vismodegib according to the intermittent schedule; all adverse events solved during the pause of treatment.

Overall, in eight of the 17 patients (47%), it was possible to re-schedule the treatment by postponing therapy for 2 weeks every 4 weeks. **Table 3** depicts their clinical characteristics. The median time to reformulation was 13.7 months (95% CI: 1.8–19.1). These patients, with an overall total duration treatment of 27.3 months (95% CI: 11.7-38.8), were all still alive at the time of the present analysis and were showing complete response (**Figure 1**). AEs resolved during the first interruption of therapy.

**Table 3:** Characteristics of the patients who switched to the vismodegib intermittent schedule.

Patient characteristics	n	%
Age (years); median (range)	77 (41–89)	
Gender:		
* Female*	5	63
* Male*	3	38
Baseline ECOG performance status:		
* 0*	3	38
* 1*	3	38
* 2*	2	25
Histology:		
* Micronodular*	3	38
* Ulcus Rodens*	3	38
* Desmoplastic*	2	25
Disease site:		
* Head*	5	63
* Inferior legs*	0	0
* Body and Head*	3	38
TNM:		
* T3N0M0*	8	100
* T4M0N1*	0	0
Comorbidities	7	88
Gorlin-Goltz syndrome	2	25
Major axis (cm)*; median (range)	0.7 (0.5–10.0)	
First treatment:		
* Surgery*	5	63
* Radiotherapy + surgery*	2	25
* Photodynamic + surgery*	1	13
* None*	0	0

*Major axis refers to each individual BCC. The smaller lesions (0.5 cm) refer to: i) a single critical lesion within multiple BCC; and ii) to single BCC at a high-risk site (e.g. ocular canthus), already treated and not amenable to therapies other than vismodegib.

In the nine remaining patients who, for any reason (AEs: n=7; disease progression: n=1; experimental protocol: n=1), did not reschedule vismodegib treatment, the median treatment duration was 5.4 months (range: 2.4–6.7). At the last assessment, four patients (44%) showed a complete response, while five patients (56%) progressed; two of them died after 1 and 3 months from disease-correlated reasons, respectively.

## Discussion

Although vismodegib is widely used for the treatment of advanced BCC, further research has been advocated on its effectiveness and treatment schedules in clinical practice (12, 13). In particular, some concerns on the use of vismodegib have been raised due to the frequent onset of AEs associated with this molecule, especially cramps and dysgeusia (16–18). The onset of these events may lead to treatment interruption and, potentially, to tumor progression.

On these bases, the description of a “field-practice” series of patients on vismodegib and the evaluation of treatment schedules aimed at allowing the continuation of vismodegib treatment also in the case of AEs is of utmost importance in the current debate on BCC therapy.

Here, we described our experience with vismodegib in a specialized cancer center. Although with all the limitations of any retrospective analysis, we reported a 100% rate of disease control at the first evaluation, confirming the effectiveness of vismodegib in clinical practice. However, this evaluation was performed, in line with our practice, only 2 months after the initiation of therapy, and three patients progressed shortly afterwards.

In line with previous literature (16–18), all the patients in our series with controlled disease showed cramps or dysgeusia over the course of therapy, requiring interruption of the standard therapy. We considered the possibility of switching to an intermittent vismodegib schedule for all these patients, consisting of 4-week courses with 2-week interruptions in between. This change in treatment schedule was possible for eight patients, given their overall favorable clinical characteristics (e.g., good performance status) and personal decisions, also taking into account the possibility of continuous caregiving by family members and the advanced age of patients.

Due to the explorative nature of our analysis, we were not able to search for any characteristics associated with the possibility of changing the treatment schedule, or to provide a definite explanation of why patients who did not change the treatment schedule showed a worse prognosis compared with those who switched to the intermittent schedule. However, some patients who did not change treatment schedule presented more advanced BCC, ulcerated and localized on the face. Moreover, those patients were elderly and/or frail subjects and could continue the treatment with difficulty (with early suspensions without schedule changes). Therefore, an early initiation of therapy should be recommended especially for older patients with multiple morbidities, in order to have a relevant impact on the course of the disease and to improve their quality of life.

We believe that proper discussion with physicians and above all the support of caregivers were important drivers for the decision not to interrupt vismodegib. All patients who switched to the intermittent schedule were able to continue vismodegib for a long period (>2 years of median time) and showed complete response to therapy.

The intermittent vismodegib schedule assessed in this pilot series may be beneficial in improving duration of treatment, allowing to maintain for a long-term treatment response, even in an elderly and frail population. Based on these preliminary findings, dedicated studies may be planned to further evaluate an intermitted schedule of vismodegib administration, also identifying the patients most suitable to this strategy.

## Data Availability Statement

The original contributions presented in the study are included in the article/supplementary material. Further inquiries can be directed to the corresponding author.

## Ethics Statement

The studies involving human participants were reviewed and approved by Humanitas Ethics Committee. The patients/participants provided their written informed consent to participate in this study.

## Author Contributions

MCT, AlS, LG, and ArS analyzed the database and wrote the article. MCT, RB, and LM managed the patients clinically and provided the iconographic material. All authors contributed to the article and approved the submitted version.

## Conflict of Interest

The authors declare that the research was conducted in the absence of any commercial or financial relationships that could be construed as a potential conflict of interest.
